# Intraventricular mass in a 49‐year‐old male

**DOI:** 10.1111/bpa.70030

**Published:** 2025-07-03

**Authors:** Connor R. Zuraski, Donald P. Pizzo, Jessica D. Schulte, Vanessa S. Goodwill

**Affiliations:** ^1^ Department of Pathology Stanford University Palo Alto California USA; ^2^ Department of Pathology University of California San Diego La Jolla California USA; ^3^ Department of Neurosciences University of California San Diego La Jolla California USA

**Keywords:** DNA methylation, intraventricular, pediatric‐type high‐grade glioma, polyimmunophenotypia, RTK1, TTF1

BOX 1 **Virtual glass slide.**

Access at https://isn‐slidearchive.org/?col=ISN&fol=Archive&file=BPA‐24‐12‐CIR‐363.R1.svs.

## 1 CLINICAL HISTORY

**FIGURE 1:**
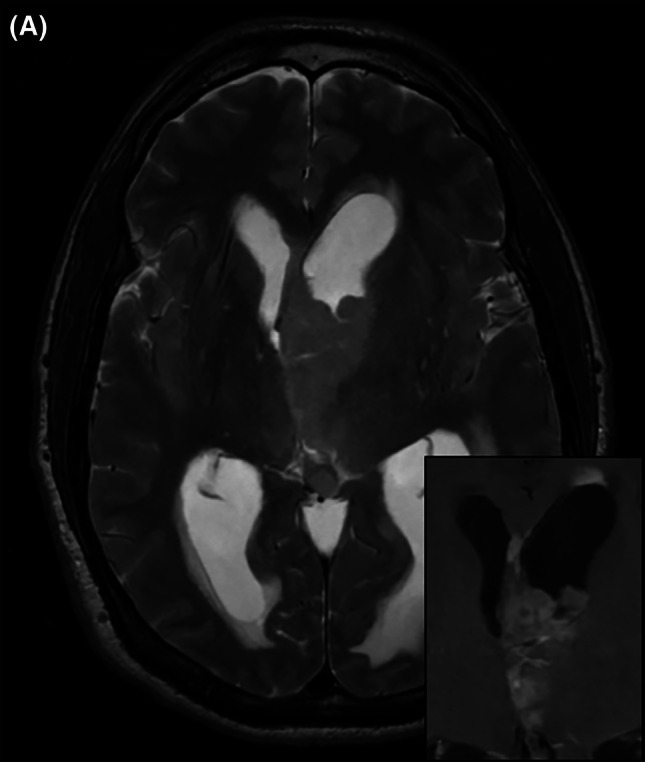
Preoperative MRI: Axial T2 3D fast‐recovery fast spin‐echo imaging demonstrates a large intraventricular mass, hydrocephalus, and transependymal edema (A). The tumor exhibits heterogeneous enhancement on T1 post‐contrast sequence (inset).

A 49‐year‐old male with no past medical history presented with 1 month of memory issues. He struggled to recall minor details and progressed to forgetting names, directions, and conversations. He developed mixed expressive/receptive aphasia, as well as a shuffling unsteady gait. Magnetic resonance imaging (MRI) of the brain showed a large intraventricular mass with restricted diffusion and heterogeneous enhancement (Figure 1). The 6.4‐cm mass was centered in the third ventricle with extension into the left lateral ventricle and cerebral aqueduct. The radiologic interpretation favored a primary central nervous system (CNS) lymphoma, with the differential including pituitary neoplasia, glioma, and germ cell tumor. CT of the chest, abdomen, and pelvis was negative for a primary source. Stereotactic biopsy was performed.

## 2 FINDINGS

**FIGURE 2:**
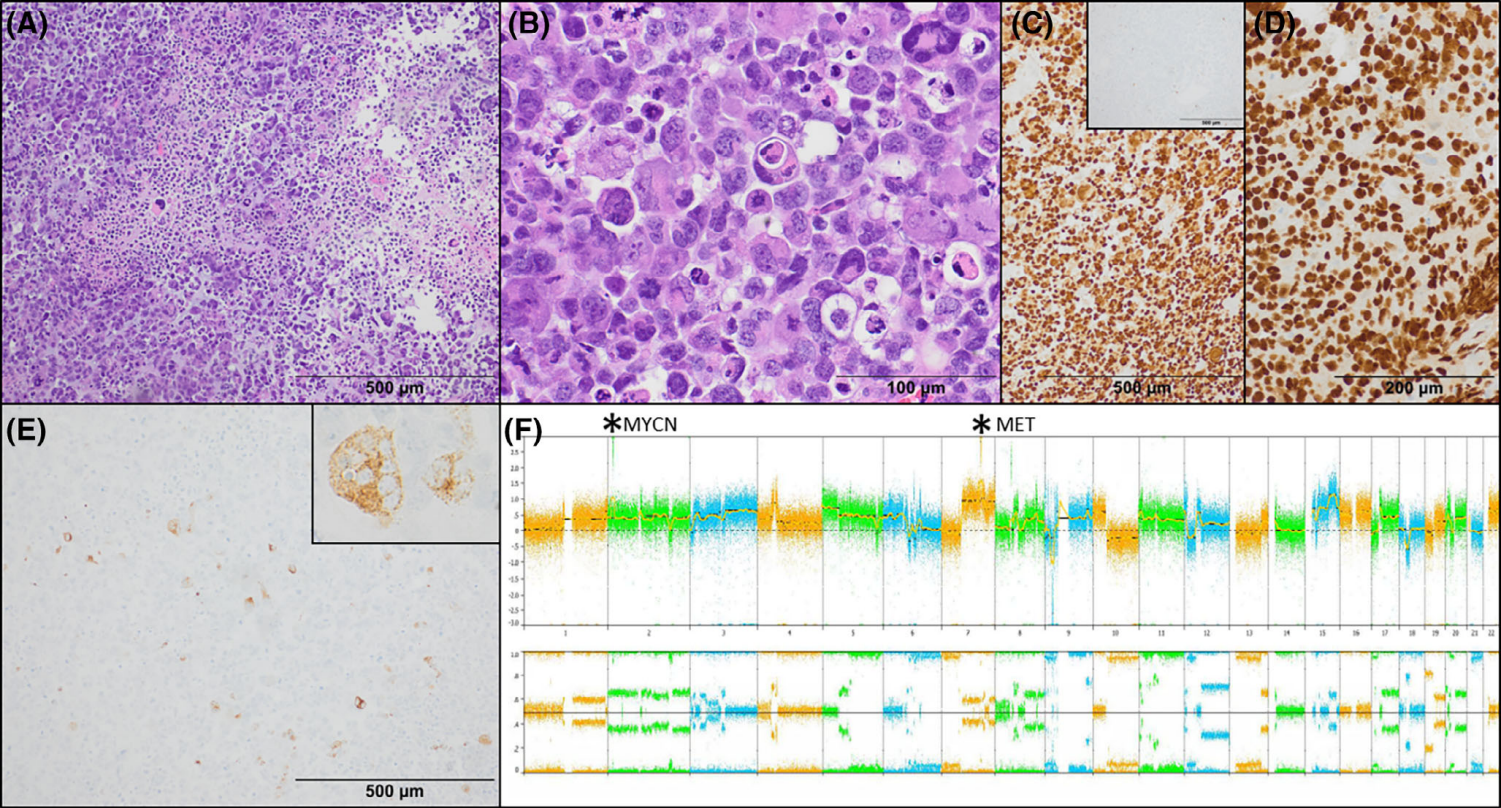
Histologic and cytogenetic features: H&E showed a densely cellular neoplasm with extreme cytologic atypia and necrosis (A and B). Immunohistochemical staining revealed strong and diffuse expression of SOX10 (C) but GFAP was negative (C, inset). Olig2 was positive on repeat biopsy 9 months later (D). A subset of tumor cells was convincingly positive for HMB45 (E). Chromosomal microarray analysis demonstrated a highly complex genome, with pertinent findings including amplifications of *MYCN* and *MET* (×25, asterisks), as well as homozygous loss of 9p encompassing *CDKN2A/B* and loss of 10q (F).

Histologic sections showed sheets of discohesive, pleomorphic tumor cells with abundant cytoplasm and bizarre nuclei with prominent nucleoli. Multinucleation, cellular cannibalism, extensive apoptosis, and necrosis were encountered. Numerous mitotic figures were seen, with up to 20 in a single high‐power (400×) field. SOX10, S100, and vimentin stains were strongly and diffusely positive. A small minority of tumor cells were convincingly positive for HMB45, and rare cells stained with SALL4, CD117, glypican‐3, myogenin, desmin, and synaptophysin. GFAP, pancytokeratin, CD45, CD30, MART1, BRAF V600E, and TTF1 (both SPT24 and 8G7G3/1 clones) were negative. INI‐1 and H3K27me3 showed retained nuclear staining (Box 1, Figure 2A–E).

Awaiting molecular analysis, a preliminary diagnosis of “high‐grade malignant neoplasm with immunohistochemical features most suggestive of melanoma” suggested the lesion could be metastatic or primary to the CNS. Next‐generation sequencing (NGS) and chromosomal microarray revealed an inactivating mutation of *TP53* and a highly complex genome with chromothripsis of 9p with homozygous loss of *CDKN2A* and *CDKN2B*, loss of heterozygosity of 17p encompassing *TP53*, amplifications of *MET, MYCN, KIT*, and *KDR* and chromothripsis of 12p13.3‐p13.2 (Figure 2F). Ultimately, DNA methylation profiling performed at the NIH/NCI showed a match to diffuse pediatric‐type high‐grade glioma (pedHGG), RTK1 subtype, subclass B, corresponding to the WHO entity of diffuse pediatric‐type high‐grade glioma, H3‐wildtype and IDH‐wildtype. The tumor matched with a score of 0.99 on the Bethesda v2 classifier and 0.65 on version 12.6 of the DKFZ classifier. Dimensionality reduction with Uniform Manifold Approximation and Projection (UMAP) also placed the tumor in the pedHGG RTK1B class.

The patient underwent chemoradiation but developed new lesions in the left caudate 9 months later. Repeat biopsy again showed negative GFAP staining, however, Olig2 immunohistochemistry, which was not available at our institution at the time of the initial biopsy, showed diffuse strong expression, further confirming glial lineage (Figure 2D). The patient died of progressive disease 15 months after presentation.

## 3 DIAGNOSIS

High‐grade glioma with methylation profile suggestive of diffuse pediatric‐type high‐grade glioma, H3‐wildtype and IDH‐wildtype, CNS WHO grade 4.

## 4 DISCUSSION

We present a challenging case of high‐grade glioma in a middle‐aged adult with a methylation profile of diffuse pediatric high‐grade glioma, IDH and H3‐wildtype, an aggressive grade 4 neoplasm with poor prognosis. Per the WHO Classification of Tumours (5th edition), essential criteria for this diagnosis include a diffuse glioma with mitotic activity occurring in a child or young adult, absence of mutations in *IDH1/2* and H3 genes, and a confirmatory methylation profile or other key molecular features [1]. On initial biopsy, GFAP negativity and aberrant expression of other markers, including the melanosomic marker HMB45, led to uncertainty regarding tumor lineage. No alterations of genes associated with metastatic or primary melanocytic lesions, such as *BRAF*, *NRAS*, or *GNAQ*, were detected, however, and strong Olig2 positivity on repeat biopsy confirmed glial origin.

This case meets published molecular requirements for diffuse pediatric glioma, H3‐ and IDH‐wildtype, although strict adherence to the WHO would preclude this diagnosis based on the patient's age. As published series describing this entity have focused on the pediatric population, cases in older adults are likely underestimated. Indeed, several methylation‐designated RTK1 pedHGGs in adult patients have been reported in a series of radiation‐induced gliomas [2].

Three molecular subtypes of H3‐ and IDH‐wildtype pedHGG have been described, each enriched for a specific gene amplification: MYCN (~50% with *MYCN* amplification), RTK1 (~33% with *PDGFRA* amplification), and RTK2 (~50% with *EGFR* amplification). Despite the presence of a *MYCN* amplification and absence of *PDGFRA* amplification, this tumor matched to the RTK1 subgroup. In a prior study delineating these three subclasses, a small subset of RTK1 tumors (2/33) demonstrated *MYCN* amplification, as in our case [1]. Additional findings in this case also occasionally reported in the RTK1 subgroup include *TP53* mutation, loss of 10q, and homozygous deletion of *CDKN2A/*B, though these findings are nonspecific and common in many high‐grade gliomas.

Tumors matching to the pedHGG RTK1 subtype often arise in the context of prior radiation or mismatch repair (MMR) defects, including sporadic defects, Lynch syndrome, or constitutional MMR deficiency [2]. Our patient had not received prior brain radiation. Immunohistochemistry for MLH1, MSH2, MSH6, and PMS2 proteins showed intact expression, and no mutations in MMR genes were identified by NGS.

In summary, we present an unusual glioma with molecular features of pedHGG in an adult patient with bizarre epithelioid morphology and absence of GFAP‐expression imparting a diagnostic challenge. In addition to broadening the morphologic spectrum of these tumors, this case highlights the need for additional studies to elucidate the full epidemiology of this recently defined tumor entity to guide future diagnostic classification.

## AUTHOR CONTRIBUTIONS

**Connor R. Zuraski:** Conceived and designed the analysis and wrote the article. **Donald P. Pizzo:** Contributed data/analysis tools (e.g. IHC). **Jessica D. Schulte:** Collected and contributed data. **Vanessa S. Goodwill:** Conceived and designed the analysis and wrote the article.

## Data Availability

Data sharing is not applicable to this article as no new data were created or analyzed in this study.
